# Loss of expression of TGF-βs and their receptors in chronic skin lesions induced by sulfur mustard as compared with chronic contact dermatitis patients

**DOI:** 10.1186/1471-5945-11-2

**Published:** 2011-01-14

**Authors:** Isa Khaheshi, Saeed Keshavarz, Abbas Ali Imani Fooladi, Majid Ebrahimi, Samaneh Yazdani, Yunes Panahi, Majid Shohrati, Mohammad Reza Nourani

**Affiliations:** 1 Genomics Division, Chemical Injury Research Center (CIRC) Baqiyatallah University of Medical Sciences, Tehran-Iran; 2 Research Center of Molecular Biology, Baqiyatallah University of Medical Sciences, Tehran-Iran; 3 School of Medicine, Tehran University of Medical Sciences, Tehran-Iran

## Abstract

**Background:**

Sulfur mustard (SM) is a blister-forming agent that has been used as a chemical weapon. Sulfur mustard can cause damage in various organs, especially the skin, respiratory system, and eyes. Generally, the multiple complications of mustard gas result from its alkalizing potency; it reacts with cellular components like DNA, RNA, proteins, and lipid membranes.

TGF-β is a multi-functional cytokine with multiple biological effects ranging from cell differentiation and growth inhibition to extracellular matrix stimulation, immunosuppression, and immunomodulation. TGF-β has 3 isoforms (TGF-β 1, 2, 3) and its signaling is mediated by its receptors: R1, R2 and intracellular Smads molecules.

TGF-β has been shown to have anti-inflammatory effects. TGF-βs and their receptors also have an important role in modulation of skin inflammation, proliferation of epidermal cells, and wound healing, and they have been implicated in different types of skin inflammatory disorders.

**Methods:**

Seventeen exposed SM individuals (48.47 ± 9.3 years), 17 chronic dermatitis patients (46.52 ± 14.6 years), and 5 normal controls (44.00 ± 14.6 years) were enrolled in this study.

Evaluation of TGF-βs and their receptors expressions was performed by semiquantitative RT-PCR. Only TGF1was analyzed immunohistochemically.

**Results:**

Our results showed significant decreases in the expression percentages of TGF-β 1, 2 and R1, R2 in chemical victims in comparison with chronic dermatitis and normal subjects and significant decreases in the intensity of R1 and R2 expressions in chemical victims in comparison with chronic dermatitis and normal controls. (P value < 0.05)

**Conclusions:**

TGF-βs and their receptors appear to have a noticeable role in chronic inflammatory skin lesions caused by sulfur mustard.

## Background

Sulfur mustard (SM) or mustard gas (bis-2-(chloroethyl)) sulfide is a blister-forming agent that was used as a chemical weapon [1] in World War I (1917) for the first time and against Iranian citizens during the Iraq Conflict (1980-1988), resulting in 100,000 chemically-injured victims[2]. Currently, one-third of these victims suffer from secondary complications [1]. SM can cause damage to various organs, especially the skin, respiratory tract, and eyes. In general, the various complications of SM are caused by its alkylating effects on cellular components such as DNA, RNA, and intramembranous proteins and lipids, resulting in metabolic and genetic damage [3-7].

In the skin, keratinocytes, particularly in the basal layer, are the main target of SM alkylation [4,8]. The major chronic skin manifestations of SM are erythema, xerosis, hypo- or hyper-pigmentation, contact dermatitis, and pruritus [9-12]. Cytokines have been shown to play a key role in acute and chronic skin inflammation, including chronic contact dermatitis due to SM [13-18]. One of these important cytokines is transforming growth factor-β (TGF-β), a 25 KD molecular weight (MW) homo-dimmer protein in its active form [19,20]. TGF-β has 3 isoforms (TGF-β 1, 2, 3), Its signaling is mediated by its transmembrane receptors, TR_1 _and TR_2_, which have serine/threonine kinase activity [21]. The intracellular signaling pathway of TGF-β is mediated by Smads molecules [22,23] that eventually enter the nucleus, bind with transcription promoters/cofactors, and regulate the transcription of DNA [24-27]. TGF-β is secreted from several cell types such as T cells, macrophages, platelets, endothelial cells, keratinocytes, and fibroblasts o[28,29]; it is a multi-functional cytokine with biological effects ranging from cell differentiation and growth inhibition to extracellular matrix stimulation, immuno-suppression, and immuno-modulation [29,30]. There have been data suggesting that the anti-inflammatory effect of TGF-β on Th1 and Th2 production and differentiation in macrophages and dendritic cells is a key issue in the skin manifestations of SM [21,27,31-38].

To evaluate the possible role of TGF-β and its receptors in chronic inflammatory skin lesions caused by SM and symptoms like pruritus, we attempted to assess the expression of TGF-β and its receptors in the skin lesions of chemical-injured victims of SM in comparison with normal controls.

## Methods

### Sampling

The subjects of this study were 17 male SM chemically-injured patients between the ages of 38 and 70 without an exposure history to toxic agents other than SM, 17 male chronic contact dermatitis patients between the ages of 20 and 68 without history of exposure to SM, and 5 healthy male participants between the ages of 21 and 58. The means and standard deviations (mean ± SD) of age were 48.47 ± 9.3, 46.52 ± 14.6 and 44.00 ± 14.6 for MS chemically-injured patients, chronic contact dermatitis patients and normal ones, respectively, and there were no significant differences in ages among the three groups (p > 0.05). The chemically-injured patients had documented histories of exposure to SM during the Iran-Iraq war (1983-88), and the chronic contact dermatitis patients had sought ambulatory medical treatment at a dermatology hospital. People with histories of addiction or topical treatment during the 48 hours before biopsy were excluded from the study. Informed consent was obtained from all the patients and normal men to be examined, and all of them were aware of the probable consequences of a skin biopsy.

The severity of the pruritus was measured subjectively by a pruritus scale (0-3). Score 0: no itching, Score 1: mild itching but no significant disturbance of daily activities; Score 2: moderate itching causing disturbance of daily activities; Score 3: severe itching causing disturbance in night sleep.

Biopsy specimens (**3 mm^2 ^size and about 25 mg weight**) were taken from pruritic plaque skin lesions under topical anesthesia with 2% lidocaine and put into trizol or **4% buffered paraformaldehyde**. The samples in trizol were transferred via -20°C rack to store at -80°C until RNA extraction, while those in formalin were placed in the refrigerator for fixation.

### RNA extraction

Total RNA was harvested in conformity with manufacturer's recommendations using trizol reagent (Invitrogen, Carlsbad, CA). Briefly described, skin biopsy specimens were homogenized in trizol by mean of an ultrasonic homogenator. After adding 200 μl chloroform (Merck, Germany) and centrifuging at 12,000 rpm, RNA containing homogenates in the aqueous phase were separated, and the same volume of isopropranol was added. To avoid contamination with proteins, the lowest fraction of the aqueous phase was not incorporated into the total RNA sample. Following centrifugation, precipitated RNA was dissolved in ethanol at 75% and centrifuged again at 1200 rpm. Isolated RNA was eluted in 20 μl RNAase-free water, and the quantity and integrity of RNA were measured by Nano Drop (ND-1000 UV - Vis spectrophotometer).

### Primer design

The primer sets for TGF-β1, TGF-β2, TGF-β receptor 1, TGF-β receptor 2, and β-actin (control gene) are shown in Table 1.

**Table 1:** Primer designs and sequences for TGF-β_1 _, TGF-β _2 _, TGF-βR_1 _and TGF-βR_2_

Product size	Annealing Time	Sequence	Name
242 bp	59	5'TCAAGCAGAGTACACACAGC3'	TGF-β_1 _Forward Primer
	
	59	5'GCACAACTCCGGTGACATC3'	TGF-β_1 _Reverse Primer

220 bp	57	5'TTGACGTCTCAGCAATGGAG3'	TGF-β_2 _Forward Primer
	
	57	5'TCAGTTACATCGAAGGAGAGC3'	TGF-β_2 _Reverse Primer

190 bp	56	5'TGCTGCAATCAGGACCATTG3'	TGF-βR_1 _Forward Primer
	
	56	5'TCCTCTTCATTTGGCACTCG3'	TGF-βR_1 _Reverse Primer

210 bp	57	5'TGCTCACCTCCACAGTGATC3'	TGF-βR_2 _Forward Primer
	
	57	5'TCTGGAGCCATGTATCTTGC3'	TGF-βR_2 _Reverse Primer

190 bp	59	5'TCATGAAGATCCTCACCGAG3'	β-actin Forward Primer
	
	59	5'TTGCCAATGGTGATGACCTG3'	β-actin Reverse Primer

**Table 2:** Severity of pruritus according to the pruritus scale in chemically-injured and chronic contact dermatitis patients

	Chemically-injured patients	Chronic contact dermatitis patients
Mild	0%	17.6%
Moderate	0%	29.4%
Severe	100%	53.0%

### cDNA synthesis and Semi quantitative RT-PCR

**Aliquots of **500 ng total RNA were reverse-transcribed to create first-strand complementary DNA by superscript III reverse-transcriptase (Invitrogen) according to the manufacturer's protocol. The resulting 1 μl of cDNA was validated with PCR in a volume of 25 ml containing 2.5 μl buffer (10x Takara), 5pm deoxynucleoside triphosphate, 0.3 μL rTq polymerase (Cinagene, Tehran, Iran) and 10 pm primer mix. PCR was carried out in the same solution with heat held at 95°c for 3 min, denaturation at 95°c for 30 sec, and annealing at 59°C, 57°C, 58°C, 56°C, 57°C, or 59°C for TGF-β1, TGF-β2, TGF-β receptor 1, TGF-β receptor 2, and β-actin, respectively, for 30 sec, extension at 72°C for 1 min (33 cycle), terminal extension at 72°c for 5 min, and a terminal hold at 4°C. PCR products were separated by 2% agarose gel electrophoresis, and the quantity of the bands was visually detectable under UV light after dying with ethidium bromide. All results were normalized with β-actin expression to compensate for differences in cDNA amount. Image analysis (using Scion Image software) was done to obtain quantitative data. (Scion Corporation, Frederick, MD)

### Immunohistochemistry

Details of the immunohistochemistry are already described elsewhere [39]. In brief, skin biopsy specimens were placed in 4% buffered paraformaldehyde for fixation.

After immersion overnight in phosphate buffer containing 30% sucrose, 20 μm thicktissue sections were cut on a cryostat and incubated with HO-1 antibody (1:200 dilution in phosphate buffer) for 12 h at 4°C. The antibody used in this study was a mouse monoclonal IgG_1 _antibody raised against recombinant TGF-β_1 _of human origin (Santa Cruz Biotechnology, Inc, USA) at a dilution of 1:200. After incubation with the primary antibody, the sections were washed with PBS and incubated with biotinylated anti-mouse secondary antibody (Santa Cruz

Biotechnology, Inc, USA). Antigen-antibody reaction sites were detectable using an ABC complex (avidin-biotinylated peroxidase complex) system (Vector Laboratory, Burlingame, CA, USA) with DAB as a substrate. For the negative control, phosphate-buffered saline (PBS) was substituted for the primary antibody.

### Statistical analysis

Data were analyzed by one-way ANOVA followed by a Bonferroni's test for multiple comparisons (using SPSS version 13). A level of P < 0.05 was considered statistically significant. All results were expressed as means ± SD.

## Results

### Clinical Findings

The pruritus scale level was severe (category 3) for all patients previously exposed to SM, while that for the chronic contact dermatitis patients varied from mild (17.6%) to moderate (29.4%) and severe (53.0%) levels (Table 2).

**Table 3:** Dermal complications due to scratching in chemically-injured and chronic contact dermatitis patients

	chemically-injured patients		chronic contact dermatitis patients	
	Yes	No	Yes	No
Excoriation	13	4	14	3
Lichenification	15	2	13	4
Erythema	14	3	14	3
Fissure	2	15	3	14

We also assessed dermal complications due to scratching in chemically injured and chronic contact dermatitis patients (Table 3).

**Table 4:** The relation between expression of TGF-β genes and severity of pruritus (1: mild, 2: moderate and 3: severe) in all patients.

Expression of TGEβ receptor 2	Expression of TGEβ receptor 1	Expression of TGF-β2	Expression of TGF-β1	Severity of pruritus	Num
+	+	-	-	3	1

-	-	-	-	3	2

-	-	-	-	3	3

-	-	-	-	3	4

-	-	-	-	3	5

-	-	-	+	3	6

-	-	-	-	3	7

-	-	-	+	3	8

-	-	-	-	3	9

-	-	-	-	3	10

-	+	-	-	3	11

-	-	-	-	3	12

-	-	-	-	3	13

-	-	-	-	3	14

+	-	+	-	3	15

-	-	-	-	3	16

-	-	-	-	3	17

+	+	+	+	3	18

+	+	+	+	2	19

+	+	-	+	3	20

+	+	+	+	1	21

+	+	+	-	3	22

-	-	-	-	3	23

-	+	-	-	2	24

+	+	-	+	1	25

+	+	+	+	3	26

-	+	+	+	2	27

-	-	+	-	3	28

-	-	-	-	2	29

-	-	-	-	3	30

+	+	+	+	3	31

-	+	-	+	1	32

-	+	-	-	2	33

+	+	-	+	3	34

There were no significant differences in excoriation, lichenification, erythema, or fissure between these two patient groups (p > 0.05).

### Molecular Biological Findings

Only two (11.7%) of the 17 SM chemically-injured patient samples expressed the TGF-β1 gene, in contrast to the chronic contact dermatitis patient samples (10 of the 17 expressing it: 58.8%) and normal controls (4 of the five expressing it: 80%), the significantly low expression rate among the three groups (p = 0.003). Only one (5.9%) of the 17 SM chemically-injured samples expressed the TGF-β2 gene, in contrast to the chronic contact dermatitis-patient samples (3 of the 17 expressing it: 47%) and normal controls (4 of the five expressing it: 80%); the expression rates among the three groups were significantly different (p = 0.002).

**Figure 1:**
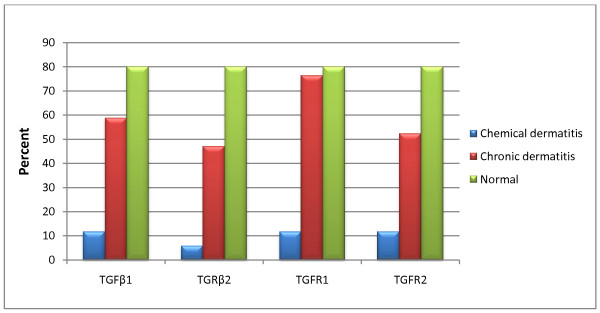
**Prevalence of expression of TGFβ1, TGFβ2, TGFR1, and TGFR2 in patients with chemical dermatitis, those with chronic dermatitis, and normal individuals**.

As the numerical comparison for TGF-β receptors reveals, only two (11.7%) of the 17 SM chemically-injured patient samples expressed the TGF-β receptor1 gene in contrast to the chronic contact dermatitis patient samples (13 of the 17 expressing it: 76.4%) and the normal controls (4 of the five expressing it: 80%); there were significant differences among the three groups (p = 0.001). Two (11.7%) of the 17 SM chemically-injured patient samples expressed the TGF-β receptor 2 gene in contrast to the chronic contact dermatitis-patient samples (9 of the 17 expressing it: 52.9%) and normal controls (4 of the five expressing it: 80%); the expression rates among the three groups were significantly different (p = 0.006) (Figure 1).

**Figure 2:**
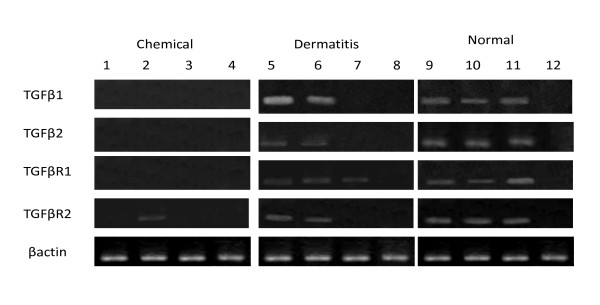
**Gene expression of TGFβ1, TGFβ2, TGFR1, TGFR2 and βactin in patients with chemical dermatitis, those with chronic dermatitis, and normal individuals measured by semi-quantitative RT-PCR. TGFβ1 = 242 bp, TGFβ2 = 220 bp, TGFR1 = 190 bp, TGFR2 = 210 bp and βactin = 190 bp**.

**Figure 3:**
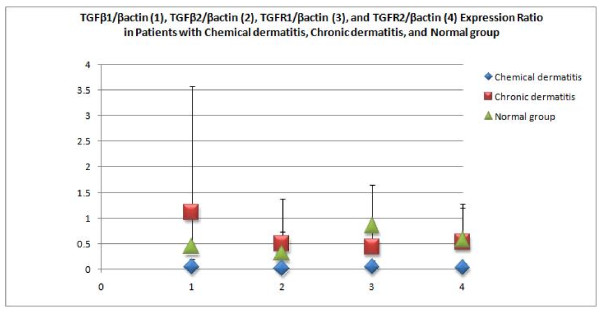
**Relative density of TGF-β genes/β-actin gene as expression ratios measured by Scion-image software in patients with chemical dermatitis, those with chronic dermatitis, and normal individuals; the error bars show standard deviation (SD)**.

With regard to the expression intensity measured by densitometer (relative density of TGF-β gene/β-actin gene), the expression intensities for TGF-β1 and -β2 genes in the chemically-injured patient samples were 0.046 ± 0.15 and 0.013 ± 0.05, respectively, in contrast to those in the chronic contact dermatitis-specimens (1.105 ± 2.46 and 0.495 ± 0.88, respectively) and that in normal ones (0.436 ± 0.74 and 0.309 ± 0.42, respectively), with insignificantly low expression levels among the three groups (p = 0.20 and p = 0.08 respectively). The expression intensities for TGF-β receptor1 and 2 genes in the positive specimens of SM chemically-injured patients were 0.046 ± 0.13 and 0.03 ± 00.08, respectively, in contrast to those in the chronic contact dermatitis patients (0.433 ± 0.45 and 0.523 ± 0.67, respectively) and in the normal controls (0.848 ± 0.79 and 0.573 ± 0.70, respectively). The expression rates among the three groups were significantly different (p = 0.001 and p = 0.01 respectively) (Figure 2, 3).

When the expression intensity and the severity of the pruritis are compared, there was a tendency for patients without genetic expression for TGF-b1 and TGF-b receptor1 to have significantly increased severity (p = 0.04 and p = 0.01 respectively), but patients without genetic expression for TGF -b2 and TGF-b receptor2 did not exhibit statistically significant increased severity (p = 0.70 and p = 0.36 respectively) (Table 4).

### Localization of TGF-β1 by immunohistochemistry

**Figure 4:**
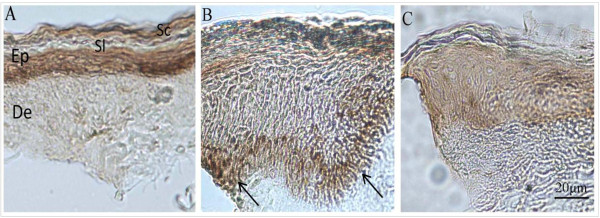
**Immunohistochemical micrograph for TGF-β1 in human skin**. A, a section from a healthy person showing discrete cell levels in the stratum corneum (Sc), stratum lucidum (Sl), epidermis (Ep) and dermis (De); TGF-β1 was strongly expressed in epidermis of healthy skin. No immunoreactivity is seen in dermis of healthy skin. B, TGF-β1 is intensely expressed in basal layer of epidermis of a chronic dermatitis patient. C, Epidermis of a chemically injured person weakly showed immunoreactivity for TGF-β1 throughout the entire epidermis.

The expression/localization of TGF-β1, as a representative of the isoforms, was examined by immunohistochemistry in the present study. In the normal skin of controls, the immunoreactivity for TGF-β1 was intense throughout all the layers of the epidermis, and no immunoreactivity is seen in the dermis (Figure 4-4A). In contrast, TGF-β1-immunoreactivity was below the significant detection level throughout both the epidermis, which was increased in thickness (Figure 4-4C), and the dermis of SM-chemically injured patients. On the other hand, in the skin of SM-independent chronic dermatitis patients, the immunoreactivity for TGF-β1 was intense in the basal layer and moderate to weak in the other cell layers of the thickened epidermis (Figure 4-4B).

## Discussion

Our results show that loss of expression of TGF-β1, TGF-β2, TGF-β receptor 1, and TGF-β receptor 2 genes in chemically injured patients is significantly more severe than in chronic contact dermatitis patients when compared with normal controls. Additionally, the frequency of severely pruritic cases is significantlyhigher in chemically injured patients than in chronic contact dermatitis patients.

Sulfur mustard and its effects on skin inflammation and the inflammatory cytokines have previously been examined in several different studies. In one animal study, the response of inflammatory cytokines was assessed in sulfur mustard-exposed mouse skin. The results emphasized the distinct role of IL-6 as a proinflammatory biomarker in sulfur mustard skin injury [13,14]. In another study, alternations of gene expression of inflammatory cytokines were detected in sulfur mustard exposed skin; the results showed significant increases in the expression of inflammatory cytokines (IL-1B, GM-CSF, IL-6) following cutaneous sulfur mustard exposure [15].

It is well known that regulatory T lymphocytes produce TGF-β and that these cells may also prepare IL-10, which, like TGF-β, has immunosuppressive effects [18]. In agreement with this fact, an interesting study has shown that overexpression of IL-10 following exposure to sulfur mustard can suppress the proinflammatory cytokines (IL-8 and IL-6) in human epidermal keratinocytes and lead to delayed cell death [40]. These findings are in agreement with our research results showing that TGF-β, like IL-10, can also have a distinct role in the modulation of skin inflammation of mustard gas. Loss of expression of TGF-βs and their receptors in the skin lesions of chemical victims may lead to retention of inflammation in the skin and chronic skin manifestations like pruritus because of lack of TGF-β control.

An animal investigation clearly reports that remarkable inflammatory lesions are detected in many organs of TGF-β1-negative mice [35]. Moreover, severe immune pathology was detected in TGF-β knockout mice [30], supporting our results.

The importance of the antiinflammatory role of TGF-β has been also emphasized in studies of signaling pathway mediators like Smad 3. These reports testify to this fact that TGF-β signaling via Smad 3 has an important role in modulation of inflammation in atopic and contact dermatitis; TGF-βs and theirs signaling mediators bridle the inflammation flares mediated by other cytokines, chemokines, and inflammatory cells. In future studies, Smad molecules should be examined as possible targets in the skin lesions of chemical victims.

In some other studies, it has been demonstrated that TGF-β has a important in wound healing; thus we see delays in wound healing in TGFβ1-knockout mice [45]. This finding suggests that loss of expression of the TGF-β family in skin lesions of mustard gas may explain the chronic skin complaints of these patients.

Various studies have checked the expression of TGF-β family and their receptors in normal skin and different regions; most of them agree that the expression of TGFβ1, 2, 3 and TGF-β R1, TGF-β R2 (as mRNA or protein levels) is detectable in human keratinocytes and layers of the skin. Our investigation also detected this expression at the mRNA and protein levels, particularly in normal samples which usually express TGF-βs and their receptors.

Matrix - Metallo proteinases (MMPs) also have a role in the inflammatory processes of sulfur mustard [50]; other research has shown that TGF-β can inhibit MMPs [29,51]. Without the inhibitory control of TGF-βs, these MMPs can be expected to continue their inflammatory effects. It has also been suggested that a lack of TGF-β may play an important role in both hyperproliferation and malignant conversion in the skin and skin tumors [52].

Paradoxically, a few studies have described proinflammatory mechanisms of TGF-β in skin pathologic conditions and its effects on chemo attraction [30,53-55]. These studies question the overall assumption that TGF-β primarily has anti-inflammatory and immuno-modulatory effects. Future investigations should clearly focus on analyzing TGF-β roles in immunopathological processes.

Finally, it is important to consider studies which have assessed some therapeutic approaches to this type of poisoning. The use of anti-oxidants and inhibitors of NF-Kappaβ has been shown to be beneficial for sulfur mustard treated human keratinocytes [56,57].

Moreover, another study has suggested that the TGF-β/Smad pathway can be useful in the treatment of atopic dermatitis [44].

Some other investigations have focused on the induction of TGF-β_1 _and TGF-β_2 _secretion by retinoic acid (isotretinoin) [58], which can lead to inhibitory effects of TGF-β on both inflammation and proliferation in the skin [59,60]. Calcipotriol (a vitamin D3 analogue) has been described to increase the secretion and activation of TGF-β1 and TGF-β2 in murine skin cells [61]. In another study, tacrolimus ointment (FK506) appeared to upregulate TGF-β release in keratinocytes as a treatment goal in dermatitis; this again reinforces an anti-inflammatory role for TGF-β in skin disorders [62,63].

These therapeutic studies may help us in future investigations targeting the TGF-β family and its signaling pathway and designed to cure or diminish the chronic skin manifestations of sulfur mustard damage, including chronic pruritus, which is resistant to common remedies.

## Conclusions

In summary, we clarified that most chemically injured patients did not express TGF-β1, TGF-β2, TGF-β receptor 1, or TGF-β receptor 2. All of this group of patients have severe pruritus as a chief complaint.

Nevertheless, detailed information about mustard gas effects on human skin, particularly at the molecular level, is very limited, and this investigation with such a small sample size cannot answer all the remaining questions. However, it can serve as a trigger for new research examining the molecular pathology of SM skin injury and thus developing new therapeutic approaches.

## Competing interests

The authors declare that they have no competing interests.

## Authors' contributions

IK carried out the molecular biology studies, alignment and drafted the manuscript, participated in the clinical examination data. SA clinical examination and taking biopsies. AAIF participated in the molecular biology analysis. ME participated in the molecular biology analysis. SY carried out the immunoassays. MS participated in the design of the study. YP participated in the design of the study. MRN as corresponding author participated in all stages of study and participated in its design and coordination and helped to draft the manuscript. All authors read and approved the final manuscript.

## Pre-publication history

The pre-publication history for this paper can be accessed here:

http://www.biomedcentral.com/1471-5945/11/2/prepub
